# Accuracy of a computer vision system for estimating biomechanical measures of body function in axial spondyloarthropathy patients and healthy subjects

**DOI:** 10.1177/02692155221150133

**Published:** 2023-01-13

**Authors:** Neil J Cronin, Maedeh Mansoubi, Erin Hannink, Benjamin Waller, Helen Dawes

**Affiliations:** 1 Neuromuscular Research Centre, Faculty of Sport and Health Sciences, University of Jyvaskyla, Jyvaskyla, Finland; 2 School of Sport and Exercise, University of Gloucestershire, Gloucester, UK; 3 Intersect@Exeter, Medical School, 171002 University of Exeter, Exeter, UK; 4 Biomedical Research Center, Medical School, Faculty of Health and Life sciences, 6397 University of Exeter, Exeter, UK; 5 Centre for Movement, Occupational and Rehabilitation Science (MOReS), 6395 Oxford Brookes University, Oxford, UK; 6 Physical Activity, Physical Education, Sport and Health Research Centre (PAPESH), Sports Science Department, School of Science and Engineering, 64401 Reykjavik University, Reykjavik, Iceland; 7 Good Boost Wellbeing limited, London, UK; 8 Oxford Health, Biomedical Research Centre, University of Oxford, Oxford, UK

**Keywords:** Artificial intelligence, physiotherapy, clinical test, telerehabilitation, remote monitoring, computer vision

## Abstract

**Objective:**

Advances in computer vision make it possible to combine low-cost cameras with algorithms, enabling biomechanical measures of body function and rehabilitation programs to be performed anywhere. We evaluated a computer vision system's accuracy and concurrent validity for estimating clinically relevant biomechanical measures.

**Design:**

Cross-sectional study.

**Setting:**

Laboratory.

**Participants:**

Thirty-one healthy participants and 31 patients with axial spondyloarthropathy.

**Intervention:**

A series of clinical functional tests (including the gold standard Bath Ankylosing Spondylitis Metrology Index tests). Each test was performed twice: the first performance was recorded with a camera, and a computer vision algorithm was used to estimate variables. During the second performance, a clinician measured the same variables manually.

**Main measures:**

Joint angles and inter-limb distances. Clinician measures were compared with computer vision estimates.

**Results:**

For all tests, clinician and computer vision estimates were correlated (*r*^2^ values: 0.360–0.768). There were no significant mean differences between methods for shoulder flexion (left: 2 ± 14° (mean ± standard deviation), *t* = 0.99, *p* < 0.33; right: 3 ± 15°, *t* = 1.57, *p* < 0.12), side flexion (left: − 0.5 ± 3.1 cm, *t* = −1.34, *p* = 0.19; right: 0.5 ± 3.4 cm, *t* = 1.05, *p* = 0.30) and lumbar flexion ( − 1.1 ± 8.2 cm, *t* = −1.05, *p* = 0.30). For all other movements, significant differences were observed, but could be corrected using a systematic offset.

**Conclusion:**

We present a computer vision approach that estimates distances and angles from clinical movements recorded with a phone or webcam. In the future, this approach could be used to monitor functional capacity and support physical therapy management remotely.

## Introduction

Clinical testing of body function plays an important role in both diagnosis and rehabilitation of common musculoskeletal conditions and is usually performed in dedicated facilities such as hospitals and community clinics. Some musculoskeletal conditions, such as axial spondyloarthropathy, require ongoing monitoring to guide self-management, so access to regular testing must be flexible and convenient. However, the need for appropriate facilities and trained staff means that testing is hindered by time and financial costs.^1^ This was further compounded by the Covid-19 pandemic, which limited peoples’ ability to travel and reduced access to musculoskeletal services, placing further strain on healthcare systems.^2^

Recent developments in the field of artificial intelligence, particularly computer vision, offer potential solutions to the difficulties of administering functional tests. For example, OpenPose ^3,4^ is an open-source algorithm trained to detect human anatomical landmarks in images, and has been used to track complex human movements from data collected with low-cost cameras.^5^ Nowadays almost all mobile phones include a camera, and mobile phone usage and literacy are extremely high, even among older adults.^6^ Numerous clinical interventions have also reported that patient acceptance of mobile phones in research is high.^7^ By combining the use of low-cost phone cameras with computer vision algorithms, it is possible to perform functional tests anywhere, and to obtain quantitative results automatically.^8^ Such a system could allow individuals to perform the tests in their own homes and without the need for direct supervision, reducing the associated costs of testing, and removing the need for dedicated facilities. This would be especially valuable for those living with chronic conditions such as axial spondyloarthropathy, who attend regular clinic appointments to measure posture and movement with a standardised test, the Bath Ankylosing Spondylitis Metrology Index (BASMI).^9^

The purpose of this study was to demonstrate the accuracy and concurrent validity of a semi-automated computer vision system that allows distance and angle parameters to be assessed from a range of common functional tests (including the modified Bath Ankylosing Spondylitis tests) recorded with a mobile phone or webcam. Accuracy was assessed by imaging objects with known dimensions using different camera positions and settings. For concurrent validity, distance/angle parameters estimated with the computer vision system were compared to the gold standard of a trained clinician.

## Methods

This study was part of a larger clinical study that aimed to examine the validity of a computer vision system to assess movement in various functional tests (ClinicalTrials.gov Identifier: NCT04895826). The reader is referred to a protocol paper for additional details.^10^ Note that fewer tests are included in the present study than in the protocol paper because during pilot testing it became clear that some test variables could not reliably be estimated using computer vision (specifically: chest expansion, standing posture and the modified Schober test). The study was approved by the Oxford Brookes University institutional ethical committee (UREC Registration No: 201429) and was conducted in accordance with the Declaration of Helsinki. The test protocol included two stages presented in detail below. Firstly, the accuracy of the computer vision system was tested using different camera positions and objects with known dimensions. The second stage involved concurrent validity testing in two participant groups, whereby the results generated by our computer vision system were compared to those of a trained clinician.

### Participants

A group of 31 healthy participants (11 males, age 35 ± 8, body mass index 27.57 ± 3.57; 20 females, age 36 ± 11, body mass index 25.00 ± 3.48) and a group of 31 participants with axial spondyloarthropathy (18 males, age 53 ± 12, body mass index 27.34 ± 3.24; 13 females, age 55 ± 14, body mass index 27.37 ± 7.43) were recruited via social media and the National Axial Spondyloarthritis Society between the 1st of March and the 25th of June 2021.

A minimum of 17 participants were required per group, assuming 1-beta = 0.90, alpha = 0.05 and effect size |*ρ*| = 0.50. Inclusion criteria for the healthy group were aged 18 or above, and healthy with no long-standing back pain. Exclusion criteria were surgery within 6 months of registration; inability to stand independently; inability to answer screening questions about participation in physical activity; serious neurological condition preventing normal movement or walking ability; and any severe medical conditions, such as cardiovascular disease.

### Computer vision system accuracy

To confirm the accuracy of our computer vision approach, we first performed three tests in a controlled setting: (1) with a camera-object distance of 2 m, we imaged a series of objects with known dimensions. Each object was also imaged at three different heights (low, mid, high) to determine whether perspective distortion affected dimension estimates. (2) We imaged the same objects at camera-object distances of 2, 3 and 4 m to determine whether this variable affected computer vision estimates of object dimensions. For tests 1 and 2, prior to each test, a calibration checkerboard was held parallel to the camera and at the same distance from the camera at which the test was to be performed. Python's OpenCV package was used to automatically detect the corners of the checkerboard, and this information combined with the known distances between checkerboard squares was used to scale all distance values from pixels to cm. (3) Since one aim of our system is to enable users to perform tests without supervision, we examined the effects of misalignment of the calibration checkerboard on computer vision estimates of object dimensions. To do this, the calibration frame was first held parallel to the camera, and then rotated in increments of 5° with respect to the camera axis up to 35°. We then quantified the effect of each calibration orientation on estimates of a known distance.

### Concurrent validity

Participants performed a series of functional tests, including a modified version of the BASMI (Table 1; Supplemental video 1), whilst standing or sitting at a fixed distance of 1.5 m from the camera. For all except one of the tests, no time limit was given and participants performed two repetitions per test. For the sit-to-stand test, they were instructed to perform five repetitions as fast as possible. In addition, for all tests except the sit-to-stand, participants repeated the movement a second time, during which an experienced certified physiotherapist (blinded to group to reduce risk of bias) manually measured variables of interest (defined in Table 1) so that these values could later be compared to the results obtained with computer vision. For a description of how the tasks were performed, see Hannink *et al*.^10^

**Table 1.** Description of the functional tests that were examined and the variables that were computed manually by a clinician and with our computer vision approach.

Test	Camera position	Measured variable(s) – computer vision	Measured variable(s) – manual
Tragus to wall	Side	Minimum horizontal distance between tragus and ipsilateral shoulder	Minimum horizontal distance between tragus and wall
Cervical rotation	Front	Change in distance between the nose and corresponding ear from the head forward position to the point of maximal cervical rotation	Peak change in the position of the tragus relative to the sternum
Shoulder flexion	Side	Peak angle between the upper arm and torso (shoulder-hip axis)	Same as for computer vision
Side flexion	Front	Minimum vertical distance between the wrist and ipsilateral knee	Same as for computer vision
Lumbar flexion	Side	Minimum vertical distance between the wrist and ipsilateral ankle	Same as for computer vision
Hip abduction	Front	Peak angle of thigh with respect to pelvis	Same as for computer vision
Hip internal rotation	Front	Peak horizontal distance between the left and right ankles	Same as for computer vision
Sit-to-stand	Side	Number of completed repetitions	Same as for computer vision

### Analysis

**Figure 1.**
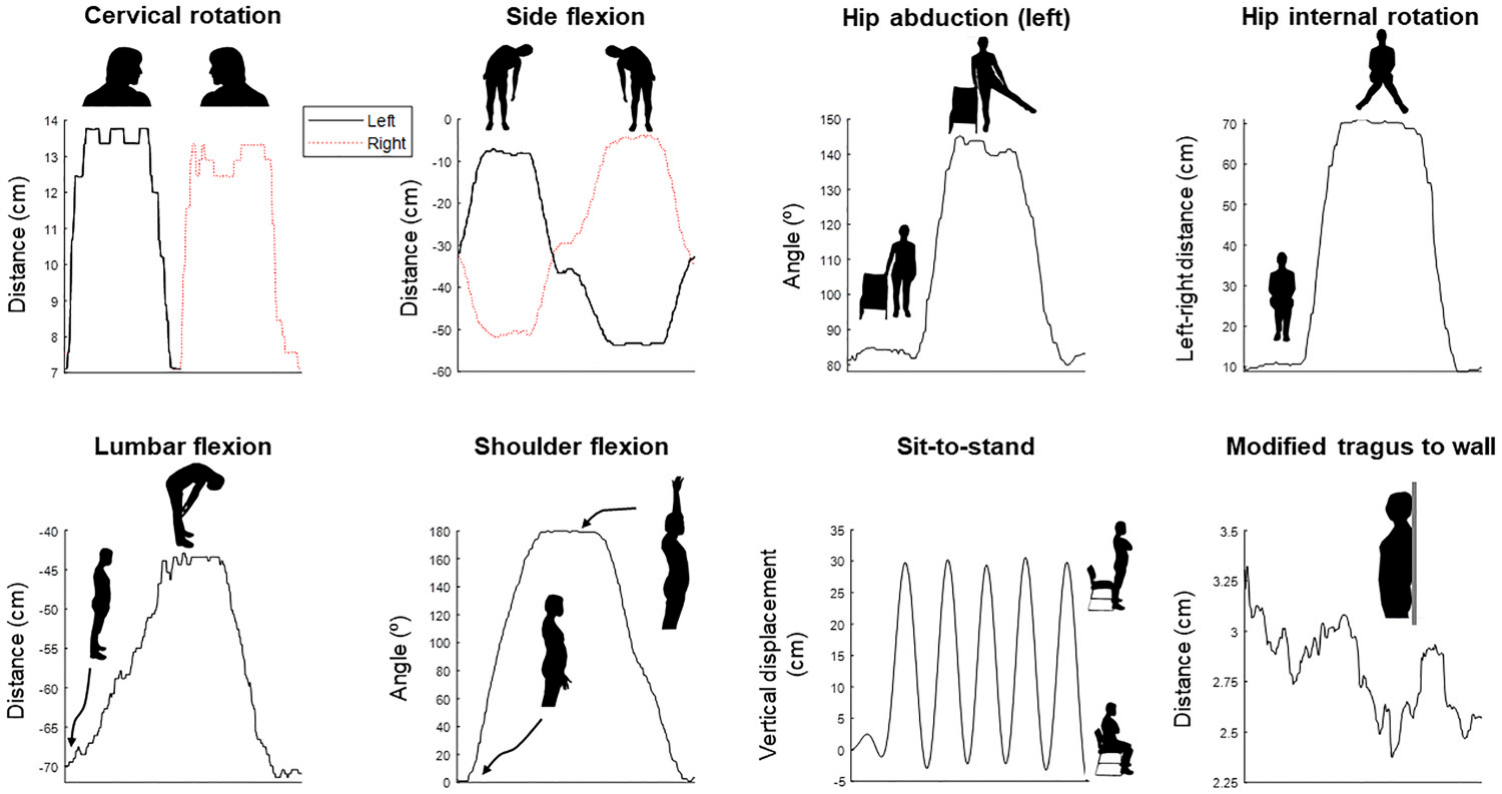
Typical output traces from the computer vision system for each test for a single participant. Movement phases are identified on each panel.

OpenPose ^3,4^ is a computer vision algorithm trained to detect key landmarks on the human body in camera images. Because it has been trained on many thousands of images, it is sufficiently robust to be used in almost any setting. For a given frame of image/video data, OpenPose returns predicted *x*,*y* coordinates for each body part and each human detected in the image. We used the *x*,*y* coordinates to compute joint angles and distances between two body parts for each test (Table 1) using Python software (Python Software Foundation, v3.8). Angles were computed using the atan2 function applied to the coordinates of two body segments. Distances were computed as either the absolute distance in the horizontal or vertical direction or using the distance formula. In both cases, distances were first computed in pixels and then translated into real-world values using the calibration procedure outlined above. For this purpose, a research assistant held up a calibration checkerboard in front of the camera prior to each test. Computer vision analysis and comparisons between methods were performed post-hoc and independently to minimise any risk of researcher bias. Typical examples of the output of our approach are shown for a single participant in Figure 1.

### Statistics

To determine computer vision system accuracy, errors between computer vision estimates and known dimensions/angles were calculated and reported as % difference. For all functional tests where scoring was continuous, manual and computer vision-derived scores were compared using Bland-Altman plots and 95% confidence intervals.^11^ Data were first tested for normality with a Shapiro-Wilk test, and data that were not normally distributed were log transformed before computing Bland-Altman plots and 95% confidence intervals, as suggested by Giavarina.^12^ Missing value analysis confirmed that missing data were randomly distributed and excluded from the comparisons. One-sample *t*-tests were used to determine whether differences between methods were significantly different to 0 (where *p* < 0.05 denotes statistical significance). Pearson's correlations between the two methods were also computed. The Pearson's *r* values were squared and effect size was interpreted as small (*r*^2^ > = 0.01), medium (*r*^2^ > = 0.09) or large (*r*^2^ > = 0.25).^13^ For the sit-to-stand test, where the outcome was discrete (number of repetitions), Mann–Whitney U was used to compare methods.

## Results

### Computer vision system accuracy

**Figure 2.**
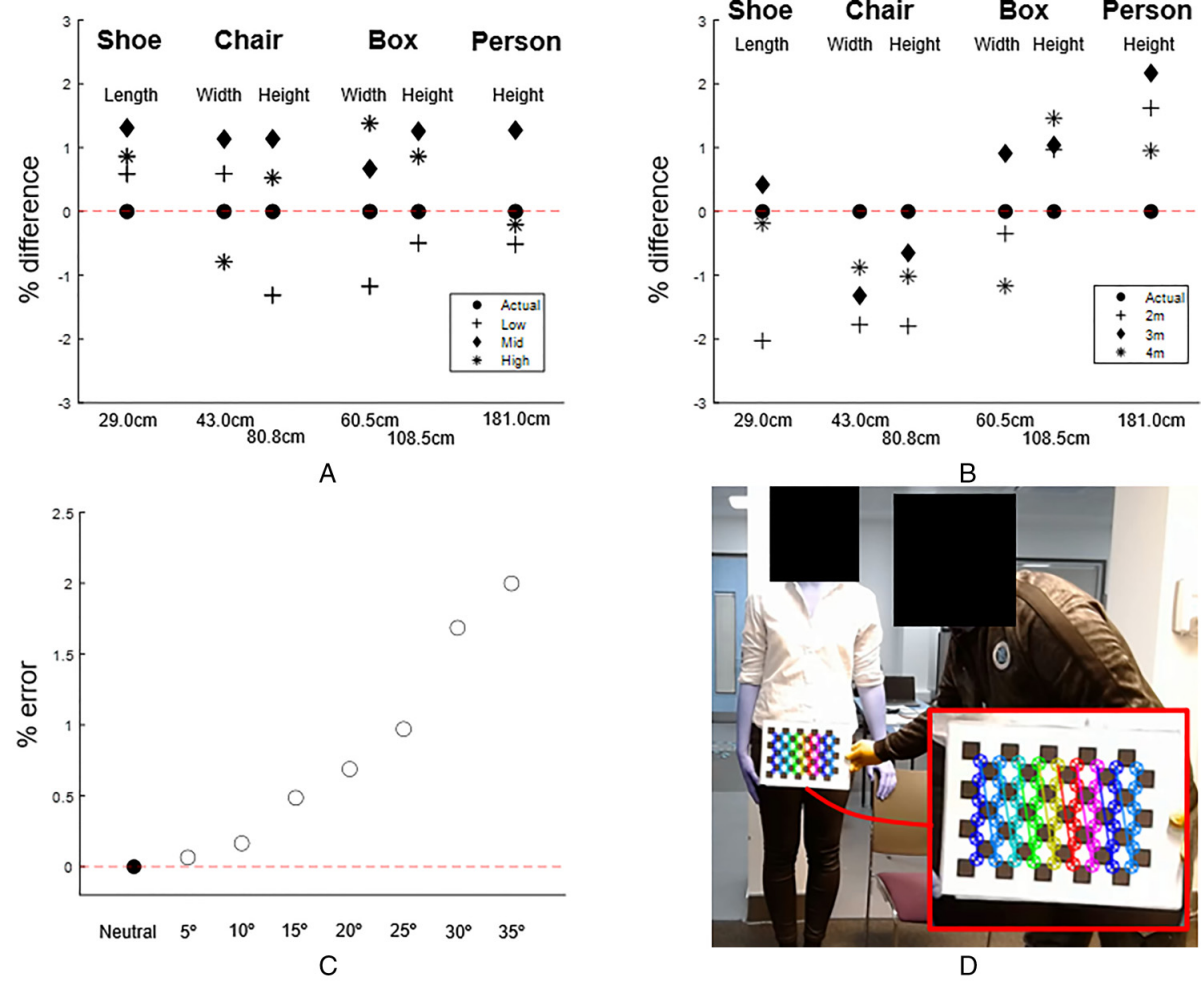
Accuracy test results. (A) Using computer vision to determine the length/width of objects with known dimensions. Positive values denote where computer vision overestimates the correct value. The correct dimensions for each object are shown below the *x*-axis. Low, Mid and High are trials where the object was positioned at different heights relative to the camera (for the person this was achieved by standing on blocks). (B) The effects of computing dimensions when the camera was placed at different distances from the object. (C) Effect of rotating the calibration checkerboard in relation to the camera on measured object dimensions. (D) Screenshot of OpenCV's processing of a calibration checkerboard, showing the automatically detected corners (enlarged in the inset).

With our computer vision approach we were able to estimate the dimensions of different sized objects to within 2% of the known dimensions, including when the objects were placed at different heights relative to the camera (Figure 2A). When examining the same objects from different distances, errors were again typically 2% or less, and there was no systematic effect of camera-object distance on the measured dimensions (Figure 2B). Scaling factors obtained from the calibration checkerboard were surprisingly robust to rotation of the checkerboard with respect to the camera (Figure 2C). Even when the checkerboard was rotated by 35°, the error for the estimation of object dimensions was only around 2%. An example of the output of automatic calibration with Python's OpenCV module is shown in Figure 2D.

## Comparison of clinician and computer vision results

**Figure 3.**
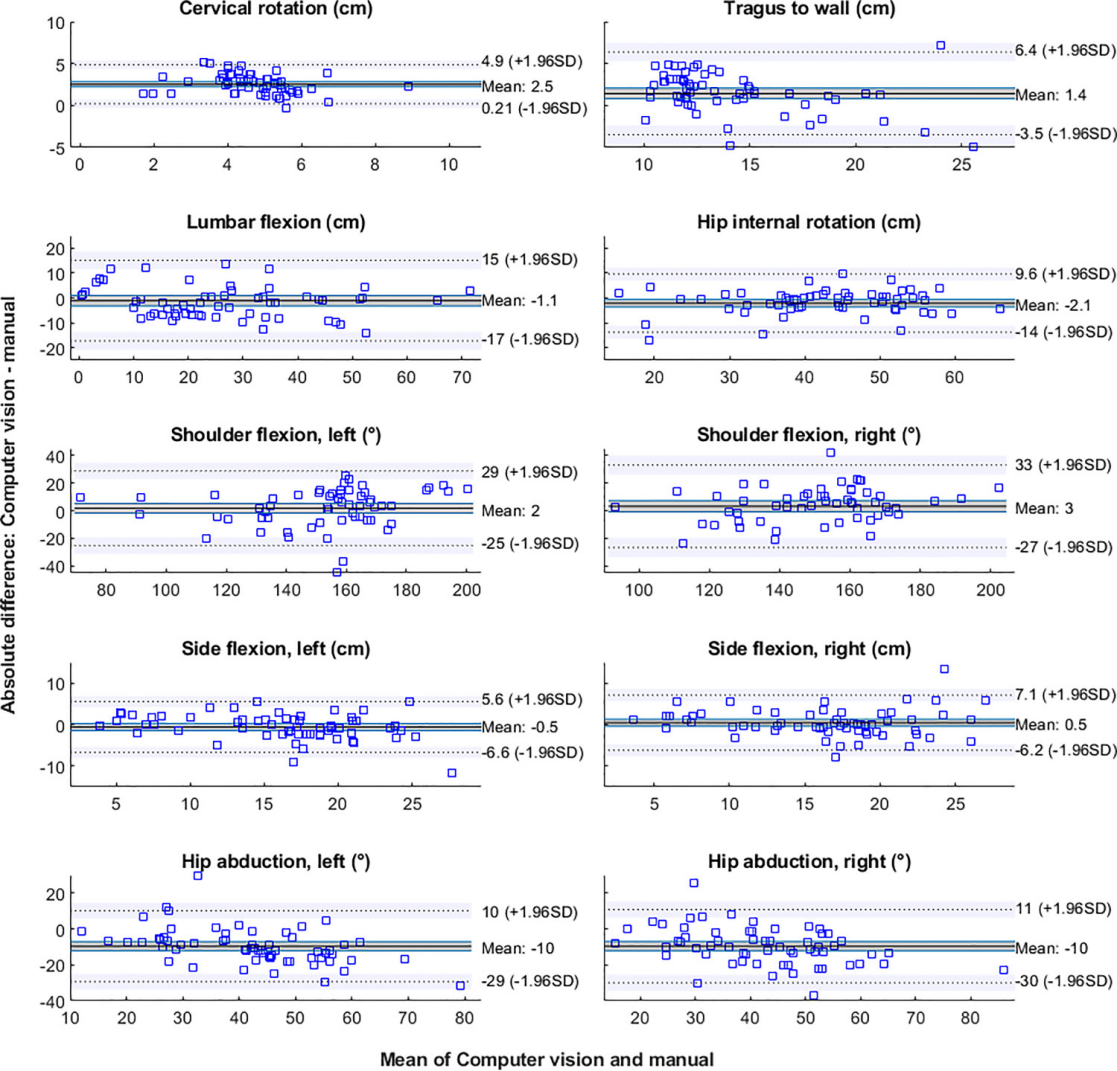
Bland-Altman plots for each test comparing manual scores to computer vision estimates. Note that the *y*-axis scale for each panel on the right is the same as for the corresponding left panel. Dotted horizontal lines show the upper and lower limits of agreement, and the surrounding shaded regions represent 95% confidence intervals of these limits. The shaded region around the mean represents the confidence interval of the mean difference between computer vision and manual estimates.

**Figure 4.**
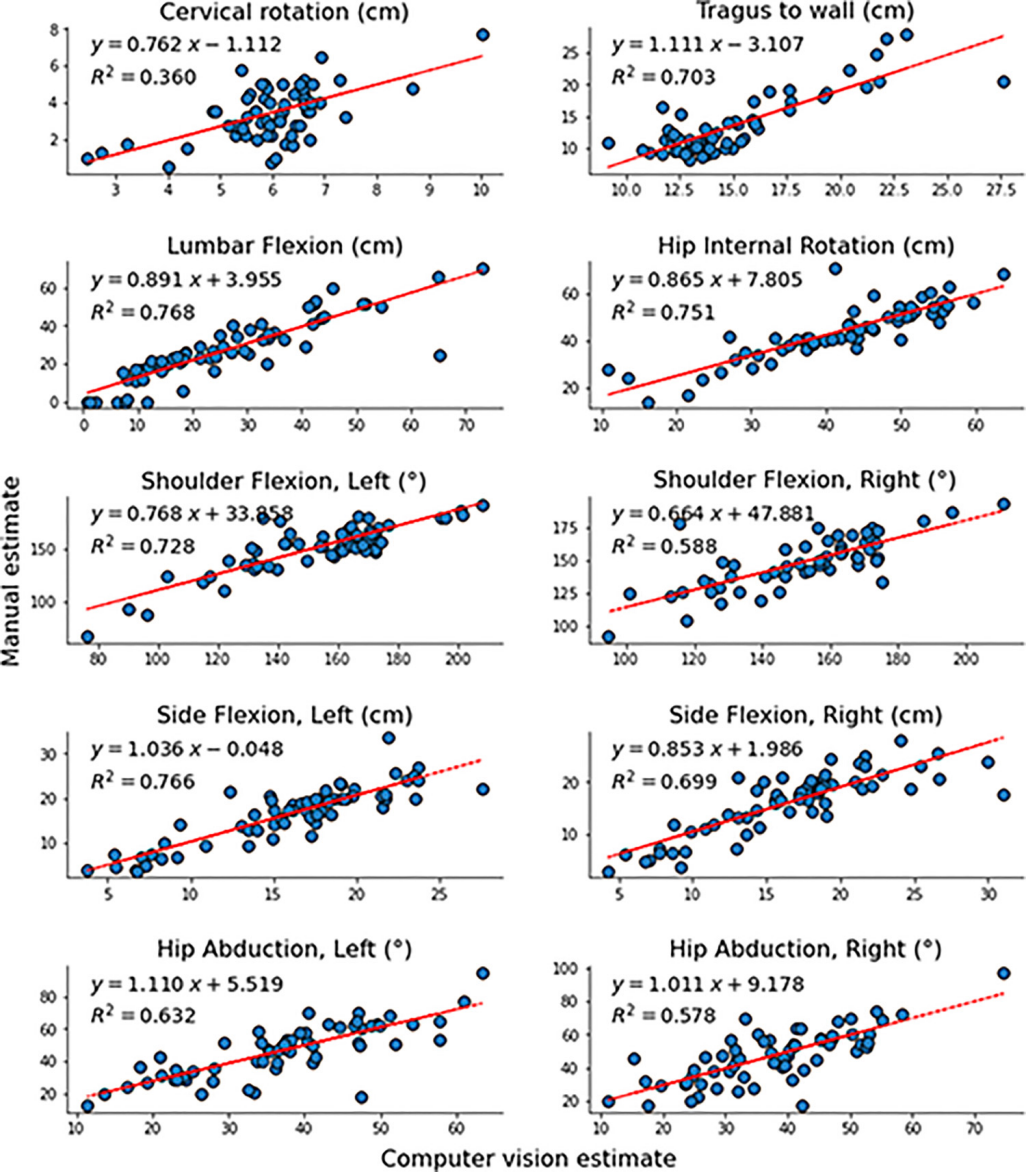
Correlation plots for each test comparing manual scores (*y*-axes) to computer vision estimates (*x*-axes).

Mean differences between methods and 95% confidence intervals are shown for all tests in Table 2. Mean differences between methods were not significant for shoulder flexion (left: 2 ± 14°, *t* = 0.99, *p* < 0.33; right: 3 ± 15°, *t* = 1.57, *p* < 0.12), side flexion (left:  − 0.5 ± 3.1 cm, *t* = −1.34, *p* = 0.19; right: 0.5 ± 3.4 cm, *t* = 1.05, *p* = 0.30) and lumbar flexion ( − 1.1 ± 8.2 cm, *t* = −1.05, *p* = 0.30). This is also evident from the Bland-Altman plots in Figure 3, where the confidence interval of the mean difference between measures always overlaps 0 for the above-mentioned tests. On the contrary, there were significant mean differences for tragus-to-wall (1.4 ± 2.5 cm, *t* = 4.52, *p* < 0.001), hip internal rotation ( − 2.1 ± 6 cm, *t* = −2.78, *p* = 0.007), cervical rotation (2.5 ± 1.2 cm, *t* = 16.81, *p* < 0.001), and hip abduction (left:  − 10 ± 10 °, *t* = −7.51, *p* < 0.001; right:  − 10 ± 10 °, *t* = −7.26, *p* < 0.001), and the confidence interval of the mean difference did not overlap 0 in the Bland-Altman plots. However, for all tests, the correlations between the estimates of the two methods represented large effect sizes (Figure 4), that is, all *r*^2^ values were >0.25.^13^ For the sit-to-stand test, there was no statistical difference between methods in the number of repetitions performed (Mann–Whitney U (*n* = 60) = 3601, *z* = −0.432, *p* = 0.666).

**Table 2.** Mean differences between methods and 95% confidence intervals for each test (except sit-to-stand where the outcome was discrete). Positive mean values denote a larger mean estimate by the computer vision system compared to clinician measures, and vice versa for negative values. One sample *t*-test results are also shown for each test, where *p* values < 0.05 denote a significant difference between the computer vision and clinician-derived means.

Test (unit)	Mean difference	SD of difference	95% CI	*t* statistic	*p* value
Tragus to wall (cm)	1.4	2.5	0.6–2.3	4.52	< 0.001
Cervical rotation (cm)	2.5	1.2	2.1–3.0	16.81	< 0.001
Shoulder flexion, left (°)	2	14	−3–7	0.99	0.33
Shoulder flexion, right (°)	3	15	−2–9	1.57	0.12
Side flexion, left (cm)	−0.5	3.1	−1.6–0.6	−1.34	0.19
Side flexion, right (cm)	0.5	3.4	−0.7–1.7	1.05	0.30
Lumbar flexion (cm)	−1.1	8.2	−4.0–1.8	−1.05	0.30
Hip abduction, left (°)	−10	10	−13–6	−7.51	< 0.001
Hip abduction, right (°)	−10	10	−13–6	−7.26	< 0.001
Hip internal rotation (cm)	−2.1	6.0	−4.2–0.0	−2.78	0.007

## Discussion

We examined accuracy and concurrent validity of a computer vision system for estimating angle and distance measures from the modified BASMI and other measures of body function. We found that the system could estimate known object dimensions to within 2% error, and was robust to changes in object height, object-camera distance and large rotations (up to 35°) of the calibration checkerboard relative to the camera. When estimating angle/distance values, the computer vision system gave statistically similar estimates to the current gold standard (manual measurements by a clinician) for shoulder flexion, side flexion, lumbar flexion and sit-to-stand. For the other tests, there were significant differences between the two methods, but the measures were still well correlated (large effect size), allowing the offset to be corrected for. These results highlight the potential of low-cost computer vision approaches for administering remote functional tests, including the modified BASMI.

Several studies have used computer vision to examine human movement in one or more tasks (for review see reference^8^). Stenum *et al*. ^14^ used OpenPose to perform gait analysis, and reported mean absolute errors in sagittal plane hip, knee and ankle angles between motion capture and OpenPose of 4.0°, 5.6° and 7.4° respectively. Similarly, when detecting specific joint locations, Nakano *et al*. ^15^ reported that of all the mean absolute errors calculated between OpenPose and 3D motion analysis, approximately 47% were <20 mm, and 80% were <30 mm. However, 10% were >40 mm, largely due to incorrect tracking by OpenPose. Kidsinski *et al*. ^5^ used OpenPose to compute joint kinematics and subsequently used this information to predict gait parameters such as walking speed with high accuracy. Schmitz *et al*. ^16^ estimated sagittal and frontal plane joint angles with a single Kinect camera and reported agreement with inclinometer measurements to within 0.5°. Our results are generally consistent with previous work, and add to the literature by examining several functional tests including those from the BASMI, as well as the inclusion of an axial spondyloarthropathy group of patients. Furthermore, we examined tests that isolate the upper body (e.g. shoulder flexion), lower body (e.g. hip abduction) and whole body movements (e.g. sit-to-stand).

Based on the mean comparisons, our computer vision estimates were correlated with those made by a clinician for all tests. However, the Bland-Altman plots allowed us to also examine individual data points, and this analysis revealed that differences between methods were sometimes large. For example, for lumbar flexion, the mean difference between estimates was 1.1 cm, but the standard deviation was 8.2 cm, and the Bland-Altman limits of agreement ranged between  − 17 and 15 cm. Similarly, for shoulder flexion, mean differences were 2° (left) and 3° (right), but standard deviations were 14° and 15° respectively, leading to limits of agreement ranging between  − 25 and 29° (left) and  − 27 and 33° (right). For all of these movements, the relatively large limits of agreement were due to a small number of extreme data points, but their existence demonstrates the need to further develop the computer vision approach (note that we deliberately did not test for or remove outliers, since this could have exaggerated the accuracy of our method). Researchers aiming to use computer vision instead of manual assessment would need to define acceptable limits of agreement *a priori*, depending on the research question and precision requirements. An additional consideration when using computer vision is the tradeoff between measurement accuracy and real-world practicality. For example, with computer vision we estimated cervical rotation differently to the clinician (see definitions in Table 1) because a single camera positioned in front of the participant cannot accurately capture the angle of rotation. This likely contributed to the significant mean difference between methods (2.5 cm), which represented about 74% of the mean manually measured distance. This could be solved by mounting a camera above the participant, or performing the test lying down, but neither of these would be practical for remote testing. Tests such as those in the BASMI were not designed for remote measurement, and this may restrict the use of fully remote systems for clinical testing to simpler, more planar tests or modified versions of common tests, as used here.

In spite of the promising computer vision results, it is logical that there was not perfect overlap between computer vision and clinician estimates. Computer vision estimates may have been influenced by lens distortion, which we did not correct for, and/or errors in the tracking of the three anatomical points needed to compute an angle. Also, OpenPose does not detect anatomical landmarks such as bones, but has been trained to detect general landmarks, which were labelled by non-experts.^17^ This likely causes some differences with respect to manual measures, where bony landmarks are often used as reference points. As already noted above, it was also not always possible to estimate parameters in the exact same way that the physiotherapist did (see Table 1 for a description of how each variable was estimated). Moreover, the physiotherapist results reported here were the average of two repetitions, whereas the computer vision system only returned the peak value from the two repetitions. Manual measures also include inherent variation and error, which could be caused by inexact placement of a goniometer or tape measure.^18^ For joint angle measures, Reissner *et al*. ^19^ have shown that this error can be up to 7° with respect to the actual angle. It should be noted that computer vision and clinician estimates were taken from separate trials, so inter-trial variability (and clinician error) may have affected our results. Previous evidence from a repeated seated reaching task performed by healthy and low back pain participants suggests that within-session reliability is moderate, with an intraclass correlation coefficient of 0.60–0.73.^20^ Similarly, lumbar displacement is highly repeatable during flexion and extension in the absence of fatigue.^21^ It therefore seems likely that inter-trial variability had only minimal effects on our findings, although we were not able to quantify this. Finally, human movement is 3-dimensional but our approach uses a single camera and is 2-dimensional, so any out-of-plane rotation would not be accounted for by the computer vision estimate. We chose movements that primarily occur in a single plane to minimise this source of error, but it cannot be completely avoided.

One challenge in this study was the need to hold up the calibration checkerboard before each trial. Although this process was quite robust, we did lose some data due to reflections on the checkerboard that made it impossible to detect the checkerboard pattern automatically, or because the checkerboard was otherwise obscured. It should be noted that this stage is only needed for distance estimates, whereas joint angles can be accurately estimated without calibration information. We did not implement our approach in real-time so that it could be used on a standard central processing unit, but real-time tracking is possible with sufficient hardware, using OpenPose or other markerless methods such as DeepLabCut.^22,23^ It is also likely that the performance of our computer vision system could be improved by imposing stricter test conditions, for example, by ensuring that the participant stays in the correct plane to minimise the effect of rotation errors on angle estimates.

## Conclusions

We demonstrate that for a range of common clinical tests of body function (including the modified BASMI) performed by populations with and without axial spondyloarthropathy, a markerless computer vision approach provides estimates of distance and angle measures that are generally statistically comparable to manual measures performed by a trained clinician. In the future, this kind of approach could be used to monitor functional capacity and to manage physical therapy remotely. Our approach is built on top of an open-source algorithm, and thus lowers potential barriers to implementing accurate movement analysis outside of a lab environment.

**Clinical messages**

- Computer vision estimates of angle/distance values were not statistically different to manual estimates by a clinician for shoulder flexion, side flexion, sit-to-stand and lumbar flexion.
- For all other tests, there were significant differences between manual and computer vision estimates, but they were still well correlated (large effect size), allowing the offset to be corrected for.
- In the future, low-cost computer vision approaches could be used to administer functional tests remotely, including the modified BASMI.

## Supplemental Material

sj-docx-1-cre-10.1177_02692155221150133 - Supplemental material for Accuracy of a computer vision system for estimating biomechanical measures of body function in axial spondyloarthropathy patients and healthy subjects Click here for additional data file. Supplemental material, sj-docx-1-cre-10.1177_02692155221150133 for Accuracy of a computer vision system for estimating biomechanical measures of body function in axial spondyloarthropathy patients and healthy subjects by Neil J Cronin, Maedeh Mansoubi, Erin Hannink, Benjamin Waller and Helen Dawes in Clinical Rehabilitation
