# Supplementary material for: Accuracy of a computer vision system for estimating biomechanical measures of body function in axial spondyloarthropathy patients and healthy subjects
Source: Clin Rehabil. 2023 Jan 13;37(8):1087–98. doi: 10.1177/02692155221150133 (PMC10291378; doi:10.1177/02692155221150133)

Supplementary figure 1. Bland-Altman plots for each test comparing manual scores to computer vision estimates. For 7 out of 10 test conditions the results presented here are identical to those in Figure 2; data from the other 3 conditions were log transformed because they were not normally distributed.


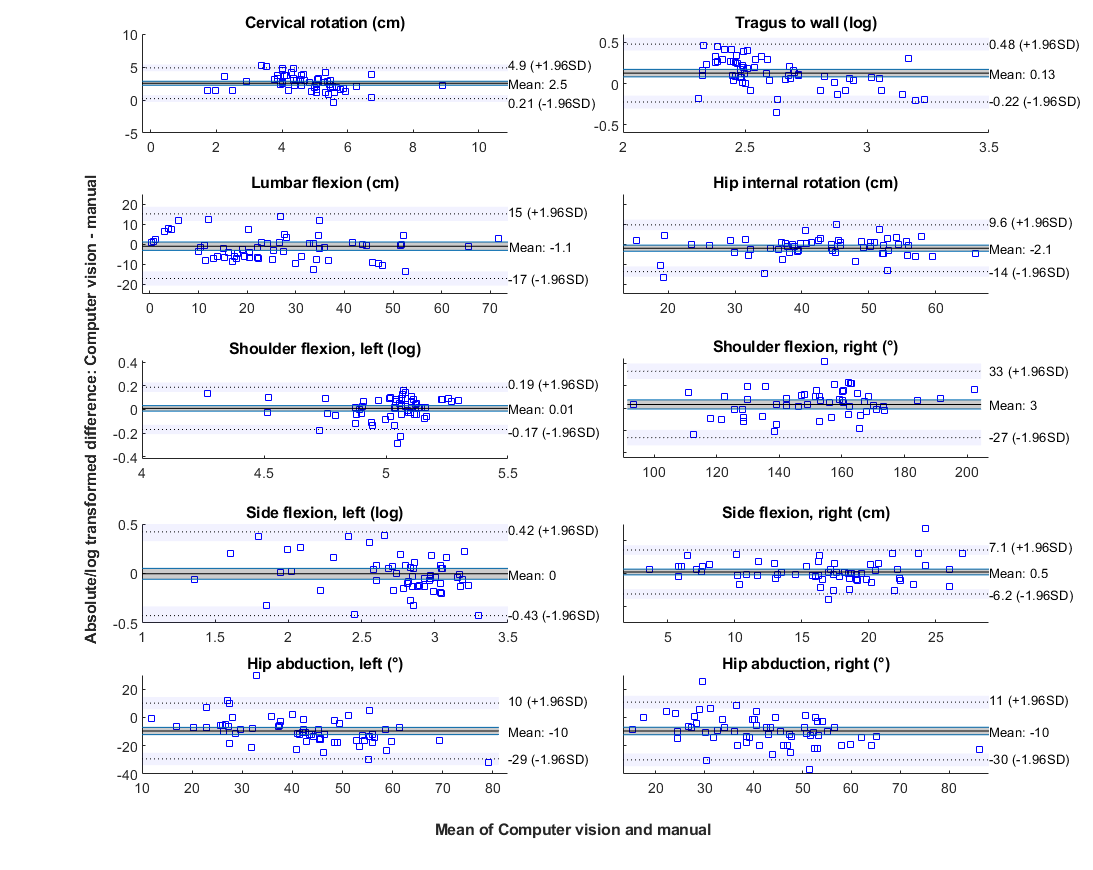

Supplement: sj-docx-1-cre-10.1177_02692155221150133 - Supplemental material for Accuracy of a computer vision system for estimating biomechanical measures of body function in axial spondyloarthropathy patients and healthy subjects [file sj-docx-1-cre-10.1177_02692155221150133.docx]
